# Review of codelists used to define hypertension in electronic health records and development of a codelist for research

**DOI:** 10.1136/openhrt-2024-002640

**Published:** 2024-04-15

**Authors:** Georgie May Massen, Philip W Stone, Harley H Y Kwok, Gisli Jenkins, Richard J Allen, Louise V Wain, Iain Stewart, Jennifer Kathleen Quint

**Affiliations:** 1 School of Public Health, Imperial College London, London, UK; 2 National Heart and Lung Institute, Imperial College London, London, UK; 3 Department of Population Health Sciences, University of Leicester, Leicester, UK; 4 NIHR Biomedical Research Centre, University of Leicester, Leicester, UK

**Keywords:** Electronic Health Records, Hypertension, Epidemiology

## Abstract

**Background and aims:**

Hypertension is a leading risk factor for cardiovascular disease. Electronic health records (EHRs) are routinely collected throughout a person’s care, recording all aspects of health status, including current and past conditions, prescriptions and test results. EHRs can be used for epidemiological research. However, there are nuances in the way conditions are recorded using clinical coding; it is important to understand the methods which have been applied to define exposures, covariates and outcomes to enable interpretation of study findings. This study aimed to identify codelists used to define hypertension in studies that use EHRs and generate recommended codelists to support reproducibility and consistency.

**Eligibility criteria:**

Studies included populations with hypertension defined within an EHR between January 2010 and August 2023 and were systematically identified using MEDLINE and Embase. A summary of the most frequently used sources and codes is described. Due to an absence of Systematized Nomenclature of Medicine Clinical Terms (SNOMED CT) codelists in the literature, a recommended SNOMED CT codelist was developed to aid consistency and standardisation of hypertension research using EHRs.

**Findings:**

375 manuscripts met the study criteria and were eligible for inclusion, and 112 (29.9%) reported codelists. The International Classification of Diseases (ICD) was the most frequently used clinical terminology, 59 manuscripts provided ICD 9 codelists (53%) and 58 included ICD 10 codelists (52%). Informed by commonly used ICD and Read codes, usage recommendations were made. We derived SNOMED CT codelists informed by National Institute for Health and Care Excellence guidelines for hypertension management. It is recommended that these codelists be used to identify hypertension in EHRs using SNOMED CT codes.

**Conclusions:**

Less than one-third of hypertension studies using EHRs included their codelists. Transparent methodology for codelist creation is essential for replication and will aid interpretation of study findings. We created SNOMED CT codelists to support and standardise hypertension definitions in EHR studies.

**WHAT IS ALREADY KNOWN ON THIS TOPIC**

- It is important to be transparent about the methods used to conduct observational research, promoting reproducibility and aiding interpretation of results.

**WHAT THIS STUDY ADDS**

- We identified codelists used to define hypertension in observational research studies, summarising commonly used codes and recommending which codes should be used. We derived Systematized Nomenclature of Medicine Clinical Terms codelists for hypertension based on current medical guidelines for hypertension management.

**HOW THIS STUDY MIGHT AFFECT RESEARCH, PRACTICE OR POLICY**

- This study provides methodology which can be reused to produce further research which looks to investigate or adjust for hypertension. We strongly urge journals to ensure that publications of observational research adhere to guidelines which promote transparency, specifically through the enforcement of ensuring codelists are freely available and attached to the publication.

## Introduction

Hypertension (high blood pressure) is a common presentation which is a leading risk factor for both stroke and coronary heart disease, it is also the largest contributor to both morbidity and mortality worldwide.^1–3^ In 2017, Public Health England estimated that 26.2% of the English population over the age of 16 were hypertensive.^4^ The significant prevalence and associations with other long-term conditions mean that hypertension is an important condition to be considered when conducting epidemiological analyses.

Patient health records are now recorded digitally in electronic health records (EHRs) and are routinely updated throughout a person’s interaction with healthcare. They contain a wealth of information that is recorded using both clinical codes and written notes (free text). Secondary uses of EHRs are epidemiological research, owing to the large quantity of clinical information, including diagnoses, tests, symptoms and prescriptions. However, the specific codes used to determine populations when using EHR data can differ, potentially altering study outcomes.

Clinical codes are alphanumeric sequences which can be used to efficiently record clinical presentations and events. There are many clinical coding languages which have slightly different structures and use cases. The International Statistical Classification of Diseases and Related Health Problems is a coding language which has been adopted across the world to record hospitalisation and cause of death, first proposed by the WHO in 1948 and subsequently implemented in the healthcare systems of a multitude of countries.^5^ Read codes have been used in primary care by the UK National Health Service since 1985, though since April 2020 their use has been phased out.^6 7^ The replacement, Systematized Nomenclature of Medicine Clinical Terms (SNOMED CT), is a highly comprehensive clinical terminology containing over 2.5 million unique terms that describe not only diagnoses, symptoms, procedures, medications and patient characteristics but also the relationships between terms, such as whether two terms relate to the same organ system. SNOMED CT is not just used in primary care settings in the UK, it is also used internationally.^8 9^

When completing epidemiological research using EHRs, the population, exposures, outcomes and covariates must all be defined using lists of clinical codes relevant to the EHR database being used.^10^ These are termed ‘codelists’ and when applied to the data will extract exposures, covariates and outcomes. Multiple different codes can be used to record the same event (especially in a terminology as comprehensive as SNOMED CT) and therefore it is common to use multiple codes and codelists to comprehensively identify factors of interest. Clinical knowledge in both the disease area as well as its clinical coding is essential when creating codelists for epidemiological research.

It has long been suggested that transparent coding and details of phenotyping should be included in observational research and has been included in guidelines; however, the rate of reporting of individual risk factors is rarely reported even though the importance has been repeatedly highlighted.^11^ The REporting of studies Conducted using Observational Routinely collected Data (RECORD) checklist, created to complement the established STROBE (The Strengthening the Reporting of Observational Studies in Epidemiology) guidelines, describes the information that should be included when using EHRs to support reproducibility and interpretability. In particular, RECORD item 6.1 states that “The methods of study populations selection (such as codes or algorithms used to identify subjects) should be listed in detail”, while RECORD item 7.1 states “A complete list of codes and algorithms used to classify exposures, outcomes, confounders, and effect modifiers should be provided. If they cannot be reported, an explanation should be provided”.^12 13^

The objective of this study was to systematically identify codelists used to define hypertension in observational studies that use EHR data and generate recommended hypertension codelists to support reproducibility and consistency of epidemiological research in hypertension.

## Methods

### Search strategy

We conducted a search of both Embase and MEDLINE using the OVID database on 14 August 2023. We included manuscripts published between 2010 and 2023, which contained the word ‘hypertension’ in the title and had at least one keyword relating to electronic healthcare records or clinical coding (table 1). We were informed by work by MacRae *et al* which derived standardised codelists for respiratory research.^14^ Our search strategy used similar terms which were defined in their study to identify observational research studies using electronic healthcare records and clinical coding.

**Table 1:** Search terms used when identifying manuscripts on the OVID platform

Condition of interest	AND	Clinical coding/EHR terms
hypertension.m_titl.		(medical records.mp) OR (electronic healthcare records.mp) OR (clinical practice research datalink.mp) OR (CPRD.mp) OR (SAIL.mp) OR (read code.mp) OR (SNOMED.mp) OR (icd 9.MP) OR (icd 10.MP) OR (icd 11.MP) OR (medcode id.mp) OR (clinical coding.mp)

EHR, electronic health record.

### Exclusion criteria

Manuscripts had to be original research articles, available in English. They could not be preprinted articles. Any manuscript investigating maternal hypertension, pulmonary hypertension or white-coat hypertension was excluded. Manuscripts that did not include codelists were excluded.

### Data extraction

For each article, GMM extracted the following information: title, journal, year of publication, EHR data source, country of EHR and availability of codes. Codelists compromising the clinical terminologies: International Classification of Diseases (ICD) version 9 (ICD 9), version 10 (ICD 10), SNOMED CT and Read (version 2) codes were exclusively extracted for this study. In addition to the manuscript, any supplementary material and links to external repositories were reviewed to identify hypertension codelists used in the study.

A second reviewer (HHYK) analysed a portion of the studies, ensuring exclusionary criteria were abided by and validated the extraction of 50 papers which contained codes used in the analysis of codelists. The objective of this work was to identify codelists used for hypertension research, no comment on the validity of codes has been provided and as a result a risk of bias assessment was not conducted.

### Analysis

The original data sources used in each publication were described to evaluate the country that the data sources (EHR database) originated from. Then we extracted the codelists used to identify hypertension in the EHR data used by the study authors. We compared extracted codelists to identify and highlight common codes used between them to identify hypertension.

### SNOMED CT codelist creation

After searching the literature, we used the information obtained to create a codelist for hypertension using the NHS (National Health Service) SNOMED CT code browser, available from Trusted Reference Update Distribution (TRUD).^15^ Using previously established methodology codelists were further developed according to medications used to manage hypertension as defined by National Institute for Health and Care Excellence (NICE) guidelines.^10 16^

Terms present in the code browser were explored, identifying codes relating to hypertension diagnosis, blood pressure readings, resistant hypertension diagnoses as well as codelists for the medications recommended in the NICE guidelines for hypertension control (ACE inhibitors, angiotensin receptor blockers, calcium channel blockers, thiazide-like diuretics, alpha blockers, beta blockers and spironolactone). As it is recommended that these drugs be prescribed either individually or as a combination, we further developed an automated script to identify the proximity of prescriptions to identify which stage of hypertension a person has at any given point in time (https://github.com/NHLI-Respiratory-Epi/Hypertension-codelists-and-definition/tree/main/Examplecode). The scripts to generate the SNOMED CT codelist and assess prescription proximity were produced using Stata V.17/MP (StataCorp, College Station, Texas, USA).

## Results

### Codelist availability

**Figure 1:**
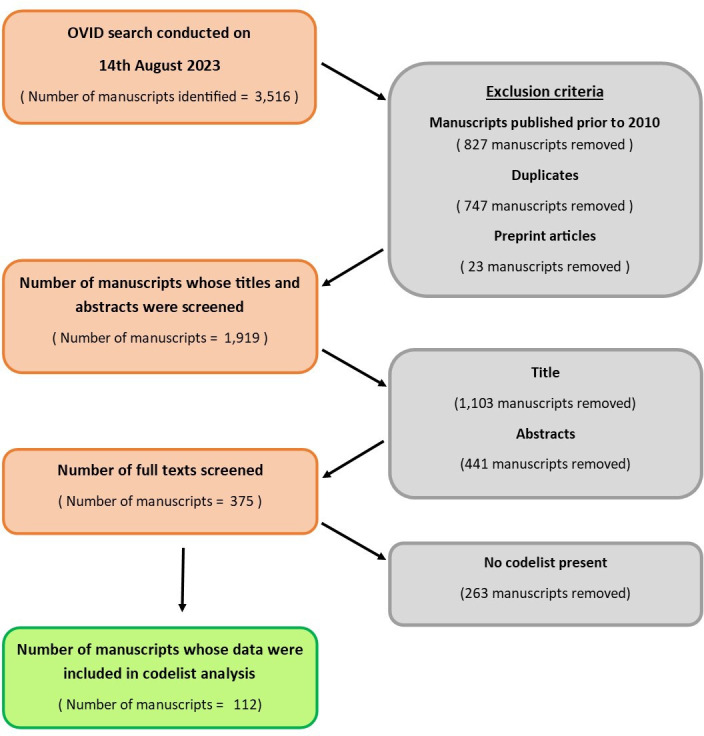
Flow chart of identification of codelists from published literature and codelist repositories.

**Figure 2:**
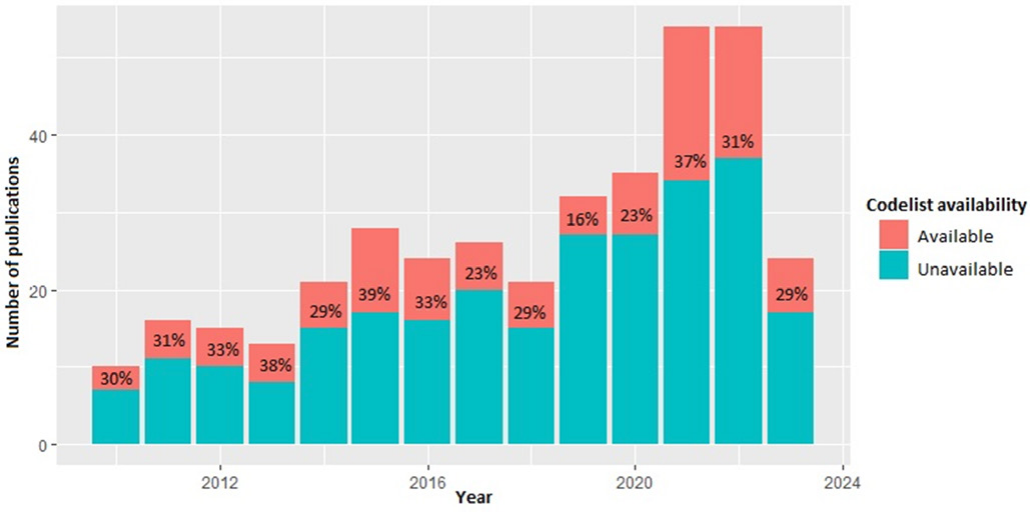
Stacked barplot demonstrating the proportion of publications per year which do and do not include codelists. Percentages show the proportion of manuscripts include codelists.

A total of 1919 articles were identified from the search of published literature (figure 1). A total of 375 were eligible for inclusion, of which 263 (71.47%) manuscripts did not state which codes were used to define hypertension within their study. 112 manuscripts detailed which codelists were used to define hypertension and were included in further evaluation. The rate of publishing codelists did not vary with respect to year (figure 2).

### Electronic healthcare records used in research

The most used data sources originated from the USA (n=53), of these the majority were databases of electronic healthcare records while some studies used data from single and multiple medical centres (online supplemental table 1). The most used database from the USA was produced by MarketScan (n=10, 18.9%). 11 of the studies used Korean data to investigate hypertension, all sourced data from the Korean National Health Insurance Service. 11 studies used data from the UK (the most used databases were the Clinical Practice Research Datalink and UK Biobank; each were used in four studies). One study included data from multiple countries.^17^ The ICD 9 and ICD 10 coding systems were the most used (n=59, n=58, respectively), seven studies used Read codes while only one study documented its use of SNOMED CT.

10.1136/openhrt-2024-002640.supp2 Supplementary data

### ICD codelists

A total of 59 manuscripts detailed the use of ICD 9 codes to define hypertension. Seven ICD 9 codes were used to identify hypertension in EHRs, these were codes 401–405, 437.2 and 362.11 (table 2). All manuscripts using this coding system included ‘401 essential hypertension’ in analyses; 27 (45.8%) of the manuscripts used this code exclusively (online supplemental file 1). The most used codelist to define hypertension was the singular 401 code for essential hypertension, while the codelist 401–405 was the second most used. We recommend that the codes 401–405 be used to identify hypertension more broadly in studies, as well as performing sensitivity analyses which only includes the data of people who have a 401 code.

10.1136/openhrt-2024-002640.supp1 Supplementary data

**Table 2:** ICD 9 codes used to define hypertension

ICD 9 code	ICD 9 code	Frequency of use
401	Essential hypertension	59
402	Hypertensive heart disease	30
403	Hypertensive renal disease	30
404	Hypertensive heart and renal disease	30
405	Secondary hypertension	23
437.2	Hypertensive encephalopathy	5
362.11	Hypertensive retinopathy	2

ICD, International Classification of Diseases.

58 manuscripts defined hypertension using ICD 10 codes, the most frequently used ICD 10 code was ‘I10 essential hypertension’ which was used 56 times (table 3). This single code was used a total of 28 times to define hypertension. The most used ICD 10 codelist included codes I10–I15, this was applied 20 times (online supplemental file 1). We recommend that the codes I10–I15 be used to identify hypertension more broadly in studies, as well as performing sensitivity analyses only including the data of people who have an I10 code.

**Table 3:** Frequency of ICD 10 code usage

ICD 10 code	ICD 10 code	Frequency of use
I10	Essential hypertension	56
I11	Hypertensive heart disease	29
I12	Hypertensive chronic kidney disease	25
I13	Hypertensive heart and chronic kidney disease	24
I15	Secondary hypertension	23
I16	Hypertensive crisis	1
I67.4	Hypertensive encephalopathy	1

ICD, International Classification of Diseases.

### Read code codelists

Seven manuscripts detailed the Read v2 codes used to define hypertension in their studies. 11 Read v2 codes were used six or more times across the seven manuscripts (table 4 and online supplemental file 1). We recommend using the Read codes present in table 3; however, it is important to accept that codelists applied may be study question specific.

**Table 4:** Most commonly used Read codes to define hypertension. NOS: not otherwise specified

Read code	Read code	Frequency of use
G202.00	Systolic hypertension	7
G201.00	Benign essential hypertension	7
G2…00	Hypertensive disease	6
G20…00	Essential hypertension	6
G200.00	Malignant essential hypertension	6
G203.00	Diastolic hypertension	6
G20z.00	Essential hypertension NOS	6
G20z.11	Hypertension NOS	6
G2z…00	Hypertensive disease NOS	6

### SNOMED CT codelists

As only one manuscript detailed the use of SNOMED CT codes to define hypertension, we cannot make comment on commonly used codes.^17^ As there appeared to be a gap in the published literature with regard to the coding of hypertension using SNOMED CT, we have developed a recommended set of codelists that can be used to define hypertension in EHRs employing SNOMED CT. These codelists are for both medications as well as clinical recording of diagnoses, informed by NICE guidelines for hypertension management.

### Codelist development

**Figure 3:**
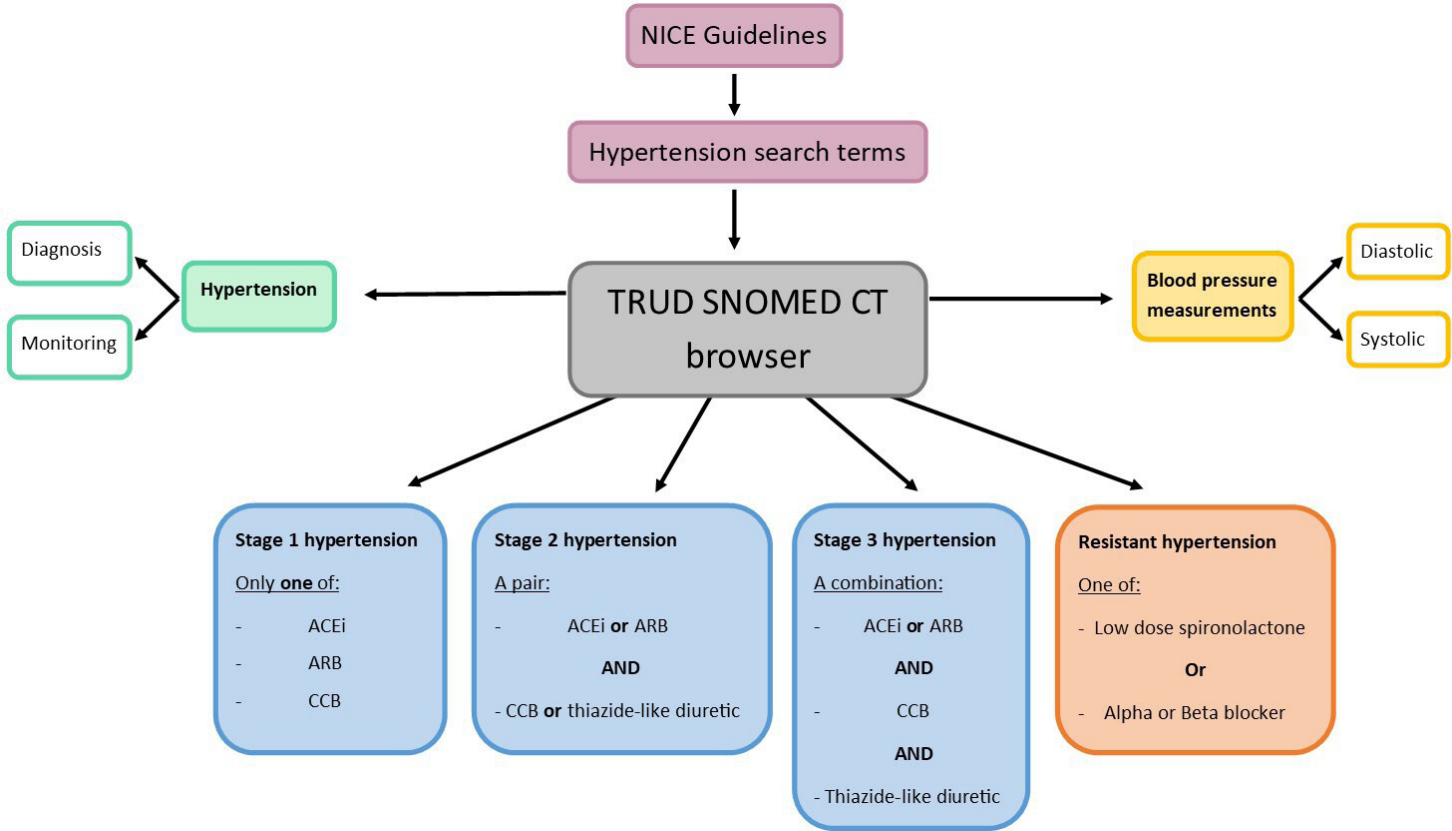
A visual summary of the codelists produced using the TRUD SNOMED CT codebrowser. ACEi, ACE inhibitors; ARB, angiotensin receptor blocker; CCB, calcium channel blocker; NICE, National Institute of Health and Care Excellence; SNOMED CT, Systematized Nomenclature of Medicine Clinical Terms; TRUD, Trusted Reference Update Distribution.

A total of 120 SNOMED CT codes were identified which indicate a diagnosis of hypertension, 132 codes were also identified which can be used to record hypertension monitoring (figure 3).

A further 22 SNOMED CT codes were identified which relating to the recording of blood pressure measurements, these were further divided into systolic and diastolic readings. The presence of these codes and values greater than 130 for the systolic codes and greater than 80 for diastolic can be used to identify high blood pressure readings and therefore hypertension.

A variety of medications can be prescribed to manage high blood pressure, we created separate codelists for ACE inhibitors (n=2,289), angiotensin receptor blockers (n=3,731), calcium channel blockers (n=4,490) and thiazide-like diuretics (n=3,536). We created code in STATA which can be used to define which stage of hypertension a person has with respect to the prescriptions they receive and the time between prescriptions, this code is hierarchically structured and is available online (https://github.com/NHLI-Respiratory-Epi/Hypertension-codelists-and-definition/blob/main/Examplecode/Stepdefinitions.do).

A singular SNOMED CT code was identified which records the presence of resistant hypertension. Using the NICE guidelines for treatment of resistant hypertension, we further developed codelists for spironolactone (n=1,156), alpha (n=1,347) and beta blockers (n=4,311) product codes, respectively, which we recommend can be used to identify a diagnosis of resistant hypertension in a person’s record; however, it is important to consider other uses of these medications.

All derived codelists are available online (https://github.com/NHLI-Respiratory-Epi/Hypertension-codelists-and-definition).

## Discussion

This work has systematically extracted codelists used to define hypertension in EHRs. Commonly used codelists were identified for EHR databases using ICD 9, ICD 10, as well as Read codes for the identification of hypertension. A significant lack of publishing of codelists was identified. This lack of reporting of the methods applied to define conditions in health records limits transparent, reproducible research. Recommended codelists were developed using the TRUD SNOMED CT browser, which can be used to define the stage of hypertension a person has and were aligned with NICE guidelines for treatment.

The RECORD statement expands on STROBE items, detailing specifically what should be present in manuscripts which use routinely collected healthcare data for observational studies. This was introduced in 2015 (during our included study period); however, since 2015 there has been no observable change in the availability of codelists with only 28.53% of all manuscripts published including codelists for hypertension. RECORD 7.1 states “A complete list of codes and algorithms used to classify exposures, outcomes, confounders, and effect modifiers should be provided. If these cannot be reported an explanation should be provided”.^12 13^ We urge authors to comply with these guidelines and to journal editors to ensure these standards are adhered to.

Few of the manuscripts that reported the codelists used to define hypertension reported the use of Read codes and only one manuscript included a SNOMED CT codelist. It is possible that this could be due to the fact that these coding systems contain many codes which use long number sequences, while the ICD uses a structured system with few trailing numbers making it easier to state codes within a manuscript, for example, it is much easier to detail ‘I10-I15’ compared with the list of 280 SNOMED CT codes which were reported by Reyes *et al.* 17

Only 29.9% of manuscripts which conducted hypertension research using routinely collected electronic healthcare records included codelists which were used to define hypertension. This is a relatively small proportion; however, it is large when compared with previous works which found that 22% of chronic obstructive pulmonary disease papers published codelists, 5% of pneumonia and acute bronchitis articles reporting used codes, respectively, and 3% of asthma articles reporting codes.^14^ It is known that codelists can bias the outcomes of a study, either through inclusion of irrelevant codes or exclusion of relevant codes^18^; hence, it is so important that studies are transparent regarding the codes used to define cohorts and covariates. The application of a codelist which misclassifies codes used to record a specific diagnosis could bias a measure of effect towards the null; therefore, poorly designed codelists could lead to important risk factors not being detected in observational studies.^19 20^ In turn, improved reporting of methodologies and codelists used in observational research would aid reproducibility of studies as well as reduce future workload (allowing more time to be spent on designing research questions rather than defining variables), therefore enabling progress in the specific field of research.^21 22^

We developed a group of recommended codelists using the TRUD SNOMED CT browser. We also provide an algorithm which can be used to define the stage of hypertension a person has. This work was based on the NICE guidelines for hypertension treatment.^23^ It is important that studies use the relevant guidelines that a clinician would be guided by in the given electronic healthcare records.

### Strengths and limitations

We systematically reviewed the available literature, providing an extensive summary of codes reported to be used to define hypertension in manuscripts which used observational data. The main weakness of this work is that there were no validation studies published on defining hypertension in EHRs and no consensus on SNOMED CT codes to be used in the published literature. We therefore developed a codelist. We did not review codelist repositories independently of publications; however, we did include codelists which had been referenced in papers and were stored in codelist repositories or other settings such as GitHub repositories. While this work provides an overview of which codelists are being used in publications, we cannot comment on the quality of these codelists. More validation studies should be done (along with a review of validation studies).

## Conclusion

The breadth of codes used to define hypertension varied between studies, leading to the creation of cohorts which will be at risk of misclassification bias. A defined set of SNOMED CT codelists relating to hypertension diagnosis and management, aligned with clinical guidelines, are recommended to support transparency of operational definitions in studies using EHR. Transparency is key in studies, and it should be a requirement that studies detail what operational definitions and corresponding codes were used to define study variables.

## Data Availability

All data relevant to the study are included in the article or uploaded as supplementary information. All works included in this analysis are referenced in the supplementary Excel file. No additional data not located within the manuscripts were used.
