# Supplementary material for: Review of codelists used to define hypertension in electronic health records and development of a codelist for research
Source: Open Heart. 2024 Apr 15;11(1):e002640. doi: 10.1136/openhrt-2024-002640 (PMC11029375; doi:10.1136/openhrt-2024-002640)
Supplement: Supplementary data [file openhrt-2024-002640supp002.pdf]

Datasource	Frequency (%)	Reference
**Australia**		
Australian Longitudinal Study on Womens Health	1 (100%)	(1)
**Canada**		
Alberta provincial data repository	2 (40%)	(2,3)
Canadian primary care sentinel surveillance network	1 (20%)	(4)
DAD	1 (20%)	(5)
MS Manitoba Database	1 (20%)	(6)
**China**		
China National Health Accounts Report 2017	1 (14%)	(7)
Multi-centre	1 (14%)	(8)
Single-centre	5 (72%)	(9–13)
**Finland**		
FinnGen Data Freeze 8	1 (100%)	(14)
**Germany**		
AOK, BKK, BARMER, TK, DAK, IKK, GEK and Knappschaft	1	(15)
German Disease Analyzer	2	(16,17)
Multi-centre	1	(18)
Regionalisierte Geburtstagsstichprobe	1	(19)
**Ghana**		
Multi-centre	1 (100%)	(20)
**Greece**		
Multi-centre	1 (100%)	(21)
**Iceland**		
Multi-centre	1 (100%)	(22)
**India**		
Multi-centre	1 (100%)	(23)
**Iran**		
Multi-centre	2 (100%)	(24,25)
**Italy**		
Brescia Health Protection Agency Database	1 (100%)	(26)
**Japan**		
Japan Medical Data Centre Database	1 (50%)	(27)
Japan National Health Insurance System	1 (50%)	(28)
**Korea**		
Korean National Health Insurance Service	10 (100%)	(29–38)
**Peru**		
MINSA Death Records	1 (100%)	(39)
**Russia**		
Multi-centre	1 (100%)	(40)
**Spain**		
SIDIAP-Q	1 (100%)	(41)
**Sweden**		
QregPV	1 (50%)	(42)
Swedish National Patient Register	1 (50%)	(43)
**Taiwan**		
National Health Insurance Database	5 (100%)	(44–48)
**Thailand**		
Civil Servants Benefit Scheme	1 (100%)	(49)
**United Kingdom**		
Clinical Practice Research Datalink	4 (40%)	(50–53)

Multi-centre	1 (10%)	(54)
The Health Improvement Network	1 (10%)	(55)
UK BioBank	4 (40%)	(56–59)
**United States of America**		
2012 Medical Expenditure Panel Survey	1 (2%)	(60)
All of Us	1 (2%)	(61)
American Manufacturing Cohort	1 (2%)	(62)
CHARYBDIS23	1 (2%)	(63)
Cigna-HealthSpring's medical claims data	1 (2%)	(64)
DC Cohort	1 (2%)	(65)
Delaware Medicaid administrative data	2 (2%)	(66,67)
EpicCare EHR	1 (2%)	(68)
Florida Cohort	1 (2%)	(69)
GE Centricity Electronic Medical Records database	2 (4%)	(70,71)
Group Health	1 (2%)	(72)
GE Healthcare	1 (2%)	(73)
HealthLNK Data Repository	1 (2%)	(74)
MarketScan Commercial Claims and Encounters Database	10 (20%)	(75–84)
IMS LifeLink Health Plan Claims Database	1 (2%)	(85)
Mississippi Vital Statistics	1 (2%)	(86)
Multi-centre	5 (10%)	(87–91)
Nationwide Inpatient Sample	4 (8%)	(92–95)
OneFlorida Data Trust	1 (2%)	(96)
Single-centre	7 (14%)	(97–103)
State Emergency Department Databases (SEDD)	1 (2%)	(104)
The Henry Ford Exercise Testing project	1 (2%)	(105)
TriNetX	1 (2%)	(106)
VA Corporate Data Warehouse	2 (4%)	(107,108)
Veterans Health Administration	1 (2%)	(109)
Veterans Integrated Service Network 20	2 (4%)	(110,111)
Wisconsin Collaborative for Healthcare Quality	1 (2%)	(112)

References

1. Navin Cristina TJ, Stewart Williams JA, Parkinson L, Sibbritt DW, Byles JE. Identification of diabetes, heart disease, hypertension and stroke in mid- and older-aged women: Comparing self-report and administrative hospital data records. *Geriatrics & Gerontology International*. 2016;16(1): 95–102. https://doi.org/10.1111/ggi.12442.

2. Quinn AE, Ronksley PE, Bresee L, Au F, Wick J, Leung AA, et al. Antihypertensive Prescribing for Uncomplicated, Incident Hypertension: Opportunities for Cost Savings. *CJC Open*. 2021;3(6): 703–713. https://doi.org/10.1016/j.cjco.2020.12.026.

3. Zongo A, Simpson S, Johnson JA, Eurich DT. Effect of a pharmacy comprehensive chronic diseases care plan on use of lipid-lowering drugs among patients with hypertension. *Journal of Managed Care & Specialty Pharmacy*. 2021;27(4): 10.18553/jmcp.2021.27.4.426. https://doi.org/10.18553/jmcp.2021.27.4.426.

4. Garies S, McBrien K, Quan H, Manca D, Drummond N, Williamson T. A data quality assessment to inform hypertension surveillance using primary care electronic medical record

data from Alberta, Canada. *BMC Public Health*. 2021;21(1): 264. https://doi.org/10.1186/s12889-021-10295-w.

5. Peng M, Chen G, Lix LM, McAlister FA, Tu K, Campbell NR, et al. Refining Hypertension Surveillance to Account for Potentially Misclassified Cases. *PLOS ONE*. 2015;10(3): e0119186. https://doi.org/10.1371/journal.pone.0119186.

6. *Rising prevalence of vascular comorbidities in multiple sclerosis: validation of administrative definitions for diabetes, hypertension, and hyperlipidemia - Ruth Ann Marrie, Bo Nancy Yu, Stella Leung, Lawrence Elliott, Patricia Caetano, Sharon Warren, Christina Wolfson, Scott B Patten, Lawrence W Svenson, Helen Tremlett, John Fisk, James F Blanchard, , for the CIHR Team in the Epidemiology and Impact of Comorbidity on Multiple Sclerosis, for the CIHR Team in the Epidemiology and Impact of Comorbidity on Multiple Sclerosis, 2012*. https://journals.sagepub.com/doi/10.1177/1352458512437814?url_ver=Z39.88-2003&rfr_id=ori:rid:crossref.org&rfr_dat=cr_pub%20%200pubmed [Accessed 2nd February 2024].

7. Liu G, Fang Q, Ji X, OuYang J, Zhu Y, Wang L, et al. Medical expenditure and its influencing factors of patients with hypertension in Shanxi Province, China: a study based on 'System of Health Accounts 2011' and multiple-layer perceptron neural network. *BMJ Open*. 2022;12(3): e048396. https://doi.org/10.1136/bmjopen-2020-048396.

8. Li S, Liu X, Li L. A Multicenter Retrospective Analysis on Clinical Effectiveness and Economic Assessment of Compound Reserpine and Hydrochlorothiazide Tablets (CRH) for Hypertension. *ClinicoEconomics and Outcomes Research: CEOR*. 2020;12: 107–114. https://doi.org/10.2147/CEOR.S231210.

9. Li C, Zhou X, Huang K, Zhang X, Gao Y. Association between Particulate Matter Pollution Concentration and Hospital Admissions for Hypertension in Ganzhou, China. *International Journal of Hypertension*. 2022;2022: 7413115. https://doi.org/10.1155/2022/7413115.

10. Chai H, Ge J, Li L, Li J, Ye Y. Hypertension is associated with osteoporosis: a case-control study in Chinese postmenopausal women. *BMC Musculoskeletal Disorders*. 2021;22(1): 253. https://doi.org/10.1186/s12891-021-04124-9.

11. Song X, Zhang Z, Zhang R, Wang M, Lin D, Li T, et al. Predictive markers of depression in hypertension. *Medicine*. 2018;97(32): e11768. https://doi.org/10.1097/MD.0000000000011768.

12. *Trends of antihypertensive agents in patients with hypertension and coronary artery disease in a tertiary hospital of China | International Journal of Clinical Pharmacy*. https://link.springer.com/article/10.1007/s11096-020-00986-6 [Accessed 2nd February 2024].

13. Diao X, Huo Y, Yan Z, Wang H, Yuan J, Wang Y, et al. An Application of Machine Learning to Etiological Diagnosis of Secondary Hypertension: Retrospective Study Using Electronic Medical Records. *JMIR Medical Informatics*. 2021;9(1): e19739. https://doi.org/10.2196/19739.

14. Ebinger JE, Kauko A, Bello NA, Cheng S, Niiranen T. Apparent treatment-resistant hypertension associated lifetime cardiovascular risk in a longitudinal national registry. *European Journal of Preventive Cardiology*. 2023;30(10): 960–968. https://doi.org/10.1093/eurjpc/zwad066.

15. *Berufsgruppen- und altersabhängige Unterschiede in der Arbeitsunfähigkeit durch häufige Herz-Kreislauf-Erkrankungen am Beispiel der essenziellen Hypertonie und des akuten Myokardinfarktes | Bundesgesundheitsblatt - Gesundheitsforschung - Gesundheitsschutz*. https://link.springer.com/article/10.1007/s00103-012-1643-8 [Accessed 2nd February 2024].

16. Jacob L, Seitz F, Kostev K. Frequency of blood pressure and estimated glomerular filtration rate monitoring in patients affected by hypertension: a retrospective study with 176 565 patients in Germany. *Blood Pressure Monitoring*. 2018;23(2): 85–90. https://doi.org/10.1097/MBP.0000000000000311.

17. Breitscheidel L, Ehlken B, Kostev K, Oberdiek MSA, Sandberg A, Schmieder RE. Real-life treatment patterns, compliance, persistence, and medication costs in patients with hypertension in Germany. *Journal of Medical Economics*. 2012;15(1): 155–165. https://doi.org/10.3111/13696998.2011.635229.

18. *Changes in blood pressure in patients with hypertension in t... : Blood Pressure Monitoring*. https://journals.lww.com/bpmonitoring/fulltext/2013/04000/changes_in_blood_pressure_in_patients_with.1.aspx [Accessed 2nd February 2024].

19. *Comparing nationwide prevalences of hypertension and depression based on claims data and survey data: An example from Germany - ScienceDirect*. https://www.sciencedirect.com/science/article/pii/S0168851016301804?via%3Dihub [Accessed 2nd February 2024].

20. Donneyong MM, Chang TJ, Pottegård A, Ankrah D, Asenso-Boadi F, Addo-Cobbiah V, et al. Prevalence and quality of antihypertensive therapy among hypertension patients enrolled in the Ghana National Health Insurance Scheme. *Pharmacoepidemiology and Drug Safety*. 2021;30(11): 1566–1575. https://doi.org/10.1002/pds.5298.

21. Notara V, Panagiotakos DB, Michalopoulou M, Kouvari M, Tsompanaki E, Verdi M, et al. Diabetes Mellitus, Hypertension and Hypercholesterolemia in Relation to the 10-Year ACS Prognosis; the GREECS Study. *Current Vascular Pharmacology*. 2016;14(3): 295–301. https://doi.org/10.2174/1570161113666150916093451.

22. Adalsteinsson SJ, Jonsson JS, Hrafnkelsson H, Thorgeirsson G, Sigurdsson EL. [Hypertension Management in Primary Care in the Capital Area of Iceland]. *Laeknabladid*. 2022;108(2): 71–78. https://doi.org/10.17992/lbl.2022.02.675.

23. *Incidence of essential hypertension in young adult males followed for over two decades - PMC*. https://www.ncbi.nlm.nih.gov/pmc/articles/PMC6309117/ [Accessed 5th February 2024].

24. Soleimani M, Jalilvand A, Ahangar H. Epidemiological Characteristic and Spatial Analysis of the Admission Rate of Hypertension in Zanjan Province, Iran. *Journal of Advances in Medical and Biomedical Research*. 2022;30(142): 443–451. https://doi.org/10.30699/jambs.30.142.443.

25. Mirahmadizadeh A, Vali M, Hassanzadeh J, Dehghani SP, Razeghi A, Azarbakhsh H. Mortality Rate and Years of Life Lost due to Hypertension in the South of Iran between 2004 and 2019: A Population-Based Study. *International Journal of Hypertension*. 2022;2022: e7759699. https://doi.org/10.1155/2022/7759699.

26. Raffetti E, Donato F, De Palma G, Leonardi L, Sileo C, Magoni M. Polychlorinated biphenyls (PCBs) and risk of hypertension: A population-based cohort study in a North Italian highly

polluted area. *The Science of the Total Environment*. 2020;714: 136660. https://doi.org/10.1016/j.scitotenv.2020.136660.

27. Kario K, Abe T, Kanegae H. Impact of pre-existing hypertension and control status before atrial fibrillation onset on cardiovascular prognosis in patients with non-valvular atrial fibrillation: A real-world database analysis in Japan. *The Journal of Clinical Hypertension*. 2019;22(3): 431–437. https://doi.org/10.1111/jch.13755.

28. Huang H, Ye Z, Nagahama I, Tazoe H, Abe Y, Aoyagi K. Impact of Hypertension, Diabetes and Dyslipidemia on Ischemic Heart Disease among Japanese: A Case-Control Study Based on National Health Insurance Medical Claims.

29. Lee H, Yano Y, Cho SMJ, Heo JE, Kim DW, Park S, et al. Adherence to Antihypertensive Medication and Incident Cardiovascular Events in Young Adults With Hypertension. *Hypertension*. 2021;77(4): 1341–1349. https://doi.org/10.1161/HYPERTENSIONAHA.120.16784.

30. Lee GB, Shin KE, Han K, Son HS, Jung JS, Kim YH, et al. Association Between Hypertension and Incident Infective Endocarditis. *Hypertension (Dallas, Tex.: 1979)*. 2022;79(7): 1466–1474. https://doi.org/10.1161/HYPERTENSIONAHA.122.19185.

31. Kim J, Kim HJ, Jeon J, Song TJ. Association between oral health and cardiovascular outcomes in patients with hypertension: a nationwide cohort study. *Journal of Hypertension*. 2022;40(2): 374. https://doi.org/10.1097/HJH.0000000000003022.

32. Byun H, Chung JH, Lee SH, Ryu J, Kim C, Shin JH. Association of Hypertension With the Risk and Severity of Epistaxis. *JAMA otolaryngology-- head & neck surgery*. 2020;147(1): 1–7. https://doi.org/10.1001/jamaoto.2020.2906.

33. *Association of periodontitis, missing teeth, and oral hygiene behaviors with the incidence of hypertension in middle-aged and older adults in Korea: A 10-year follow-up study - PubMed*. https://pubmed.ncbi.nlm.nih.gov/35446990/ [Accessed 16th November 2023].

34. Kim HC, Cho SMJ, Lee H, Lee HH, Baek J, Heo JE, et al. Korea hypertension fact sheet 2020: analysis of nationwide population-based data. *Clinical Hypertension*. 2021;27(1): 8. https://doi.org/10.1186/s40885-021-00166-2.

35. Kim HC, Lee H, Lee HH, Seo E, Kim E, Han J, et al. Korea hypertension fact sheet 2021: analysis of nationwide population-based data with special focus on hypertension in women. *Clinical Hypertension*. 2022;28(1): 1. https://doi.org/10.1186/s40885-021-00188-w.

36. Oh SH, Lee SJ, Park J. Precision Medicine for Hypertension Patients with Type 2 Diabetes via Reinforcement Learning. *Journal of Personalized Medicine*. 2022;12(1): 87. https://doi.org/10.3390/jpm12010087.

37. Kim YG, Han KD, Choi JI, Yung Boo K, Kim DY, Oh SK, et al. Impact of the Duration and Degree of Hypertension and Body Weight on New-Onset Atrial Fibrillation: A Nationwide Population-Based Study. *Hypertension (Dallas, Tex.: 1979)*. 2019;74(5): e45–e51. https://doi.org/10.1161/HYPERTENSIONAHA.119.13672.

38. Woo HG, Chang Y, Lee JS, Song TJ. Tooth loss is associated with an increased risk of hypertension: A nationwide population-based cohort study. *PloS One*. 2021;16(6): e0253257. https://doi.org/10.1371/journal.pone.0253257.

39. Herrera-Añazco P, Atamari-Anahui N, Ccorahua-Rios MS, Amaya E. National trends in age-standardized mortality attributable to hypertension in Peru. *Jornal Brasileiro de Nefrologia*. 2021;43(3): 417–421. https://doi.org/10.1590/2175-8239-JBN-2020-0009.

40. *Structure of comorbidity in urban population with essential hypertension in the clinical practice of a local general practitioner | Russian Open Medical Journal*. https://romj.org/2022-0307 [Accessed 6th February 2024].

41. Foguet-Boreu Q, Violán C, López Jiménez T, Pons-Vigués M, Rodríguez-Blanco T, Valderas JM, et al. Pharmacological control of diabetes and hypertension comorbidity in the elderly: A study of “real world” data. *Primary Care Diabetes*. 2017;11(4): 348–359. https://doi.org/10.1016/j.pcd.2017.03.007.

42. Bager JE, Mourtzinis G, Andersson T, Nåtman J, Rosengren A, Björck S, et al. Trends in blood pressure, blood lipids, and smoking from 259 753 patients with hypertension in a Swedish primary care register: results from QregPV. *European Journal of Preventive Cardiology*. 2022;29(1): 158–166. https://doi.org/10.1093/eurjpc/zwab087.

43. Stenberg E, Cao Y, Marsk R, Sundbom M, Jernberg T, Näslund E. Association between metabolic surgery and cardiovascular outcome in patients with hypertension: A nationwide matched cohort study. *PLoS Medicine*. 2020;17(9): e1003307. https://doi.org/10.1371/journal.pmed.1003307.

44. Chuang SY, Yu Y, Sheu WHH, Tsai YT, Liu X, Hsiung CA, et al. Association of short-term use of nonsteroidal anti-inflammatory drugs with stroke in patients with hypertension. *Stroke*. 2015;46(4): 996–1003. https://doi.org/10.1161/STROKEAHA.114.007932.

45. *Reverse Epidemiology of Hypertension and Cardiovascular Death in the Hemodialysis Population | Hypertension*. https://www.ahajournals.org/doi/10.1161/01.hyp.0000154895.18269.67 [Accessed 6th February 2024].

46. Yen FS, Wei JCC, Shih YH, Hsu CC, Hwu CM. The Risk of Nephropathy, Retinopathy, and Leg Amputation in Patients With Diabetes and Hypertension: A Nationwide, Population-Based Retrospective Cohort Study. *Frontiers in Endocrinology*. 2021;12: 756189. https://doi.org/10.3389/fendo.2021.756189.

47. Hung CY, Wang KY, Wu TJ, Hsieh YC, Huang JL, Loh EW, et al. Resistant Hypertension, Patient Characteristics, and Risk of Stroke. *PLOS ONE*. 2014;9(8): e104362. https://doi.org/10.1371/journal.pone.0104362.

48. Huang PH, Xirasagar S, Chen JH, Cheng YF, Kuo NW, Lin HC. Absence of Association of Tinnitus With Pre-existing Hypertension: A Population-based Study. *The Annals of Otology, Rhinology, and Laryngology*. 2023;132(7): 756–762. https://doi.org/10.1177/00034894221115756.

49. Tatsanavivat P, Thavornpitak Y, Pongchaiyakul C. Comparative effectiveness of three national healthcare schemes in Thailand: in-hospital medical expenses for diabetes and hypertension in 2010. *Journal of the Medical Association of Thailand = Chotmaihet Thangphaet*. 2012;95 Suppl 7: S254-261.

50. Costello RE, Yimer BB, Roads P, Jani M, Dixon WG. Glucocorticoid use is associated with an increased risk of hypertension. *Rheumatology*. 2021;60(1): 132–139. https://doi.org/10.1093/rheumatology/keaa209.

51. Sinnott SJ, Smeeth L, Williamson E, Douglas IJ. Trends for prevalence and incidence of resistant hypertension: population based cohort study in the UK 1995-2015. *BMJ*. 2017;358: j3984. https://doi.org/10.1136/bmj.j3984.

52. Lemp JM, Nuthanapati MP, Bärnighausen TW, Vollmer S, Geldsetzer P, Jani A. Use of lifestyle interventions in primary care for individuals with newly diagnosed hypertension, hyperlipidaemia or obesity: a retrospective cohort study. *Journal of the Royal Society of Medicine*. 2022;115(8): 289–299. https://doi.org/10.1177/01410768221077381.

53. Lay-Flurrie SL, Sheppard JP, Stevens RJ, Mallen C, Heneghan C, Hobbs FDR, et al. Impact of Changes to National Hypertension Guidelines on Hypertension Management and Outcomes in the United Kingdom. *Hypertension*. 2020;75(2): 356–364. https://doi.org/10.1161/HYPERTENSIONAHA.119.13926.

54. Mahdi A, Armitage LC, Tarassenko L, Watkinson P. Estimated Prevalence of Hypertension and Undiagnosed Hypertension in a Large Inpatient Population: A Cross-sectional Observational Study. *American Journal of Hypertension*. 2021;34(9): 963–972. https://doi.org/10.1093/ajh/hpab070.

55. Peng M, Chen G, Kaplan GG, Lix LM, Drummond N, Lucyk K, et al. Methods of defining hypertension in electronic medical records: validation against national survey data. *Journal of Public Health (Oxford, England)*. 2016;38(3): e392–e399. https://doi.org/10.1093/pubmed/fdv155.

56. Zhang S, Qian ZM, Chen L, Zhao X, Cai M, Wang C, et al. Exposure to Air Pollution during Pre-Hypertension and Subsequent Hypertension, Cardiovascular Disease, and Death: A Trajectory Analysis of the UK Biobank Cohort. *Environmental Health Perspectives*. 2023;131(1): 17008. https://doi.org/10.1289/EHP10967.

57. Huang J, Yang T, Gulliver J, Hansell AL, Mamouei M, Cai YS, et al. Road Traffic Noise and Incidence of Primary Hypertension: A Prospective Analysis in UK Biobank. *JACC. Advances*. 2023;2(2): None. https://doi.org/10.1016/j.jacadv.2023.100262.

58. Larvin H, Kang J, Aggarwal VR, Pavitt S, Wu J. The additive effect of periodontitis with hypertension on risk of systemic disease and mortality. *Journal of Periodontology*. 2022;93(7): 1024–1035. https://doi.org/10.1002/JPER.21-0621.

59. He J, Zhang S, Qiu Y, Liu F, Liu Z, Tan J, et al. Ulcerative colitis increases risk of hypertension in a UK biobank cohort study. *United European Gastroenterology Journal*. 2023;11(1): 19–30. https://doi.org/10.1002/ueg2.12351.

60. Shao H, Mohammed MU, Thomas N, Babazadeh S, Yang S, Shi Q, et al. Evaluating Excessive Burden of Depression on Health Status and Health Care Utilization Among Patients With Hypertension in a Nationally Representative Sample From the Medial Expenditure Panel Survey (MEPS 2012). *The Journal of Nervous and Mental Disease*. 2017;205(5): 397–404. https://doi.org/10.1097/NMD.0000000000000618.

61. Chandler PD, Clark CR, Zhou G, Noel NL, Achilike C, Mendez L, et al. Hypertension prevalence in the All of Us Research Program among groups traditionally underrepresented in medical research. *Scientific Reports*. 2021;11(1): 12849. https://doi.org/10.1038/s41598-021-92143-w.

62. Ferguson JM, Costello S, Neophytou AM, Balmes JR, Bradshaw PT, Cullen MR, et al. Night and rotational work exposure within the last 12 months and risk of incident hypertension. *Scandinavian journal of work, environment & health*. 2019;45(3): 256–266. https://doi.org/10.5271/sjweh.3788.

63. Reyes C, Pistillo A, Fernández-Bertolín S, Recalde M, Roel E, Puente D, et al. Characteristics and outcomes of patients with COVID-19 with and without prevalent hypertension: a multinational cohort study. *BMJ open*. 2021;11(12): e057632. https://doi.org/10.1136/bmjopen-2021-057632.

64. Abughosh SM, Wang X, Serna O, Henges C, Masilamani S, Essien EJ, et al. A Pharmacist Telephone Intervention to Identify Adherence Barriers and Improve Adherence Among Nonadherent Patients with Comorbid Hypertension and Diabetes in a Medicare Advantage Plan. *Journal of Managed Care & Specialty Pharmacy*. 2016;22(1): 63–73. https://doi.org/10.18553/jmcp.2016.22.1.63.

65. Mallipeddi VP, Levy M, Byrne M, Monroe A, Happ LP, Moeng LR, et al. Evaluation of New Hypertension Guidelines on the Prevalence and Control of Hypertension in a Clinical HIV Cohort: A Community-Based Study. *AIDS research and human retroviruses*. 2023; https://doi.org/10.1089/AID.2022.0063.

66. *Association of Area Deprivation With Primary Hypertension Diagnosis Among Youth Medicaid Recipients in Delaware | Cardiology | JAMA Network Open | JAMA Network*. https://jamanetwork.com/journals/jamanetworkopen/fullarticle/2802470 [Accessed 6th February 2024].

67. *Impact of Health Literacy on Medication Adherence Among Black Medicaid Beneficiaries with Hypertension in Delaware: A Cross-Sectional Study - PMC*. https://www.ncbi.nlm.nih.gov/pmc/articles/PMC10125397/ [Accessed 6th February 2024].

68. Banerjee D, Chung S, Wong EC, Wang EJ, Stafford RS, Palaniappan LP. Underdiagnosis of hypertension using electronic health records. *American journal of hypertension*. 2012;25(1): 97–102. https://doi.org/10.1038/ajh.2011.179.

69. Xu Y, Chen X, Wijayabahu A, Zhou Z, Yu B, Spencer EC, et al. Cumulative HIV Viremia Copy-Years and Hypertension in People Living with HIV. *Current HIV research*. 2020;18(3): 143–153. https://doi.org/10.2174/1570162X18666200131122206.

70. Chopra I, Kamal KM, Candrilli SD, Kanyongo G. Association between obesity and therapeutic goal attainment in patients with concomitant hypertension and dyslipidemia. *Postgraduate Medicine*. 2014;126(1): 66–77. https://doi.org/10.3810/pgm.2014.01.2726.

71. Chopra I, Kamal KM. Factors associated with therapeutic goal attainment in patients with concomitant hypertension and dyslipidemia. *Hospital Practice (1995)*. 2014;42(2): 77–88. https://doi.org/10.3810/hp.2014.04.1106.

72. Unmuessig V, Fishman PA, Vrijhoef HJM, Elissen AMJ, Grossman DC. Association of Controlled and Uncontrolled Hypertension With Workplace Productivity. *Journal of Clinical Hypertension (Greenwich, Conn.)*. 2016;18(3): 217–222. https://doi.org/10.1111/jch.12648.

73. Biskupiak JE, Kim J, Phatak H, Wu D. Prevalence of High-Risk Cardiovascular Conditions and the Status of Hypertension Management Among Hypertensive Adults 65 Years and Older in the United States: Analysis of a Primary Care Electronic Medical Records Database. *The*

Journal of Clinical Hypertension. 2010;12(12): 935–944. https://doi.org/10.1111/j.1751-7176.2010.00362.x.

74. Garg G, Tedla YG, Ghosh AS, Mohottige D, Kolak M, Wolf M, et al. Supermarket Proximity and Risk of Hypertension, Diabetes, and CKD: A Retrospective Cohort Study. *American Journal of Kidney Diseases: The Official Journal of the National Kidney Foundation*. 2023;81(2): 168–178. https://doi.org/10.1053/j.ajkd.2022.07.008.

75. Gatwood J, Hohmeier KC, Shuvo S, Mikaiel D, Kovesdy CP. Alignment of diagnosis and pharmacy claims data coding of medication adherence among patients with diabetes or hypertension. *Journal of Managed Care & Specialty Pharmacy*. 2021;27(4): 10.18553/jmcp.2021.27.4.497. https://doi.org/10.18553/jmcp.2021.27.4.497.

76. Desai R, Park H, Brown JD, Mohandas R, Pepine CJ, Smith SM. Comparative Safety and Effectiveness of Aldosterone Antagonists Versus Beta-Blockers as Fourth Agents in Patients With Apparent Resistant Hypertension. *Hypertension (Dallas, Tex.: 1979)*. 2022;79(10): 2305–2315. https://doi.org/10.1161/HYPERTENSIONAHA.122.19280.

77. Baker-Goering MM, Howard DH, Will JC, Beeler Asay GR, Roy K. Association Between Self-Reported Hypertension and Antihypertensive Medication Use and Cardiovascular Disease–Related Events and Expenditures Among Patients Diagnosed With Hypertension. *Public Health Reports*. 2019;134(5): 493–501. https://doi.org/10.1177/0033354919864363.

78. *Trends in Antihypertensive Medication Use Among US Patients With Resistant Hypertension, 2008 to 2014 | Hypertension*. https://www.ahajournals.org/doi/full/10.1161/hypertensionaha.116.08128 [Accessed 16th November 2023].

79. Hanselin MR, Saseen JJ, Allen RR, Marrs JC, Nair KV. Description of Antihypertensive Use in Patients With Resistant Hypertension Prescribed Four or More Agents. *Hypertension*. 2011;58(6): 1008–1013. https://doi.org/10.1161/HYPERTENSIONAHA.111.180497.

80. Thompson LA, Saseen JJ, O'Bryant CL, Allen RR, Nair KV. Claims analysis of hypertension occurrence, severity changes and patterns of antihypertensive use in cancer patients receiving vascular endothelial growth factor inhibitors. *Journal of Oncology Pharmacy Practice*. 2015;21(4): 258–267. https://doi.org/10.1177/1078155214530177.

81. Macnair A, Nankivell M, Murray ML, Rosen SD, Appleyard S, Sydes MR, et al. Healthcare systems data in the context of clinical trials - A comparison of cardiovascular data from a clinical trial dataset with routinely collected data. *Contemporary Clinical Trials*. 2023;128: 107162. https://doi.org/10.1016/j.cct.2023.107162.

82. Francis BH, Song X, Andrews LM, Purkayastha D, Princic N, Sedgley R, et al. Progression to type 2 diabetes, healthcare utilization, and cost among pre-diabetic patients with or without comorbid hypertension. *Current Medical Research and Opinion*. 2011;27(4): 809–819. https://doi.org/10.1185/03007995.2011.554806.

83. Chen S, Macaulay D, Swallow E, Diener M, Farooqui S, Xie J, et al. Real-world adherence and persistence associated with nebivolol or hydrochlorothiazide as add-on treatment for hypertension. *Current Medical Research and Opinion*. 2014;30(4): 637–643. https://doi.org/10.1185/03007995.2013.864267.

84. Chen S, Macaulay D, Swallow E, Diener M, Farooqui S, Xie J, et al. Real-world adherence and persistence associated with nebivolol or hydrochlorothiazide as add-on treatment for hypertension. *Current Medical Research and Opinion*. 2014;30(4): 637–643. https://doi.org/10.1185/03007995.2013.864267.

85. Fonseca V, Sharma PP, Shah M, Deedwania P. Risk of new-onset diabetes mellitus associated with beta-blocker treatment for hypertension. *Current Medical Research and Opinion*. 2011;27(4): 799–807. https://doi.org/10.1185/03007995.2011.555477.

86. Mendy VL, Rowell-Cunsolo T, Bellerose M, Vargas R, Zhang L, Enkhmaa B. Temporal Trends in Hypertension Death Rate in Mississippi, 2000-2018. *American Journal of Hypertension*. 2021;34(9): 956–962. https://doi.org/10.1093/ajh/hpab068.

87. Blair IV, Steiner JF, Hanratty R, Price DW, Fairclough DL, Daugherty SL, et al. An investigation of associations between clinicians' ethnic or racial bias and hypertension treatment, medication adherence and blood pressure control. *Journal of General Internal Medicine*. 2014;29(7): 987–995. https://doi.org/10.1007/s11606-014-2795-z.

88. *Characteristics of resistant hypertension in a large, ethnically diverse hypertension population of an integrated health system - PubMed*. https://pubmed.ncbi.nlm.nih.gov/24079679/ [Accessed 6th February 2024].

89. Selby K, Michel M, Gildengorin G, Karliner L, Pramanik R, Fontil V, et al. Disparities in Hypertension Control Across and Within Three Health Systems Participating in a Data-Sharing Collaborative. *Journal of the American Board of Family Medicine: JABFM*. 2018;31(6): 897–904. https://doi.org/10.3122/jabfm.2018.06.180166.

90. Dumitrescu L, Ritchie MD, Denny JC, El Rouby NM, McDonough CW, Bradford Y, et al. Genome-wide study of resistant hypertension identified from electronic health records. *PloS One*. 2017;12(2): e0171745. https://doi.org/10.1371/journal.pone.0171745.

91. *Identifying patients with hypertension: a case for auditing electronic health record data - PubMed*. https://pubmed.ncbi.nlm.nih.gov/22737097/ [Accessed 6th February 2024].

92. von Drygalski A, Kolaitis NA, Bettencourt R, Bergstrom J, Kruse-Jarres R, Quon DV, et al. Prevalence and risk factors for hypertension in hemophilia. *Hypertension (Dallas, Tex.: 1979)*. 2013;62(1): 209–215. https://doi.org/10.1161/HYPERTENSIONAHA.113.01174.

93. Polgreen LA, Suneja M, Tang F, Carter BL, Polgreen PM. Increasing trend in admissions for malignant hypertension and hypertensive encephalopathy in the United States. *Hypertension (Dallas, Tex.: 1979)*. 2015;65(5): 1002–1007. https://doi.org/10.1161/HYPERTENSIONAHA.115.05241.

94. Movahed MR, Sattur S, Hashemzadeh M. Independent association between type 2 diabetes mellitus and hypertension over a period of 10 years in a large inpatient population. *Clinical and Experimental Hypertension (New York, N.Y.: 1993)*. 2010;32(3): 198–201. https://doi.org/10.3109/10641960903254539.

95. Movahed MR, Lee JZ, Lim WY, Hashemzadeh M, Hashemzadeh M. Strong independent association between obesity and essential hypertension. *Clinical Obesity*. 2016;6(3): 189–192. https://doi.org/10.1111/cob.12139.

96. Smith SM. Hypertension in Florida: Data From the OneFlorida Clinical Data Research Network. *Preventing Chronic Disease*. 2018;15. https://doi.org/10.5888/pcd15.170332.

97. *An Observational Study of Hypertension and Thromboembolism Among Transgender Patients Using Gender-Affirming Hormone Therapy - PMC*. https://www.ncbi.nlm.nih.gov/pmc/articles/PMC7173689/ [Accessed 6th February 2024].

98. Stewart IJ, Sosnov JA, Snow BD, Batou A, Howard JT, Janak JC, et al. Hypertension after injury among burned combat veterans: A retrospective cohort study. *Burns: Journal of the International Society for Burn Injuries*. 2017;43(2): 290–296. https://doi.org/10.1016/j.burns.2016.10.005.

99. Bartels CM, Johnson H, Voelker K, Thorpe C, McBride P, Jacobs EA, et al. Impact of Rheumatoid Arthritis on Receiving a Diagnosis of Hypertension Among Patients with Regular Primary Care. *Arthritis care & research*. 2014;66(9): 1281–1288. https://doi.org/10.1002/acr.22302.

100. Walia HK, Griffith SD, Thompson NR, Moul DE, Foldvary-Schaefer N, Mehra R. Impact of Sleep-Disordered Breathing Treatment on Patient Reported Outcomes in a Clinic-Based Cohort of Hypertensive Patients. *Journal of Clinical Sleep Medicine : JCSM : Official Publication of the American Academy of Sleep Medicine*. 2016;12(10): 1357–1364. https://doi.org/10.5664/jcsm.6188.

101. Sun J, McNaughton CD, Zhang P, Perer A, Gkoulalas-Divanis A, Denny JC, et al. Predicting changes in hypertension control using electronic health records from a chronic disease management program. *Journal of the American Medical Informatics Association: JAMIA*. 2014;21(2): 337–344. https://doi.org/10.1136/amiajnl-2013-002033.

102. Sessoms J, Reid K, Williams I, Hinton I. Provider Adherence to National Guidelines for Managing Hypertension in African Americans. *International Journal of Hypertension*. 2015;2015: 498074. https://doi.org/10.1155/2015/498074.

103. Ware KS, Chidume T, Chou C. Social determinants of health and preventable emergency department patient encounters among adults with hypertension. *Public Health Nursing (Boston, Mass.)*. 2023;40(1): 171–174. https://doi.org/10.1111/phn.13152.

104. Shimada YJ, Tsugawa Y, Iso H, Brown DFM, Hasegawa K. Association of bariatric surgery with risk of acute care use for hypertension-related disease in obese adults: population-based self-controlled case series study. *BMC Medicine*. 2017;15(1): 161. https://doi.org/10.1186/s12916-017-0914-5.

105. Juraschek SP, Blaha MJ, Whelton SP, Blumenthal R, Jones SR, Keteyian SJ, et al. Physical Fitness and Hypertension in a Population at Risk for Cardiovascular Disease: The Henry Ford ExercIse Testing (FIT) Project. *Journal of the American Heart Association*. 3(6): e001268. https://doi.org/10.1161/JAHA.114.001268.

106. Stapff M, Hilderbrand S. First-line treatment of essential hypertension: A real-world analysis across four antihypertensive treatment classes. *Journal of Clinical Hypertension (Greenwich, Conn.)*. 2019;21(5): 627–634. https://doi.org/10.1111/jch.13531.

107. Salem RM, Pandey B, Richard E, Fung MM, Garcia EP, Brophy VH, et al. The VA Hypertension Primary Care Longitudinal Cohort: Electronic medical records in the post-genomic era. *Health Informatics Journal*. 2010;16(4): 274–286. https://doi.org/10.1177/1460458210380527.

108. Peralta CA, Frigaard M, Rolon L, Seal K, Tuot D, Senyak J, et al. Screening for CKD To Improve Processes of Care among Nondiabetic Veterans with Hypertension: A Pragmatic Cluster-Randomized Trial. *Clinical journal of the American Society of Nephrology: CJASN*. 2020;15(2): 174–181. https://doi.org/10.2215/CJN.05050419.

109. Min L, Ha JK, Hofer TP, Sussman J, Langa K, Cushman WC, et al. Validation of a Health System Measure to Capture Intensive Medication Treatment of Hypertension in the Veterans Health Administration. *JAMA Network Open*. 2020;3(7): e205417. https://doi.org/10.1001/jamanetworkopen.2020.5417.

110. Melzer AC, Uman J, Au DH. Adherence to Oral Medications for Hypertension and Diabetes in Veterans with Comorbid Airflow Limitation. *Annals of the American Thoracic Society*. 2015;12(6): 831–837. https://doi.org/10.1513/AnnalsATS.201501-001OC.

111. Raghavan S, Ho YL, Kini V, Rhee MK, Vassy JL, Gagnon DR, et al. Association Between Early Hypertension Control and Cardiovascular Disease Incidence in Veterans With Diabetes. *Diabetes Care*. 2019;42(10): 1995–2003. https://doi.org/10.2337/dc19-0686.

112. Ho AK, Thorpe CT, Pandhi N, Palta M, Smith MA, Johnson HM. Association of anxiety and depression with hypertension control: a US multidisciplinary group practice observational study. *Journal of Hypertension*. 2015;33(11): 2215–2222. https://doi.org/10.1097/HJH.0000000000000693.
